# Optimization of Ultrahigh-Throughput Screening Assay for Protein Engineering of d-Allulose 3-Epimerase

**DOI:** 10.3390/biom12111547

**Published:** 2022-10-24

**Authors:** Zhanzhi Liu, Shuhan Liu, Jingyi Jia, Liuxin Wang, Feng Wang, Xiaoyue Pan, Jing Wu, Sheng Chen

**Affiliations:** 1 State Key Laboratory of Food Science and Technology, Jiangnan University, 1800 Lihu Avenue, Wuxi 214122, China; 2 Key Laboratory of Industrial Biotechnology Ministry of Education, School of Biotechnology, Jiangnan University, 1800 Lihu Avenue, Wuxi 214122, China; 3 International Joint Laboratory on Food Safety, Jiangnan University, 1800 Lihu Avenue, Wuxi 214122, China

**Keywords:** d-allulose 3-epimerase, directed evolution, biosensor-based screening assay, optimization

## Abstract

d-Allulose is the corresponding epimer of d-fructose at the C-3 position, which exhibits a similar taste and sweetness to sucrose. As a low-calorie sweetener, d-allulose has broad application prospects in the fields of medicine, food, and so on. Currently, the production method of d-allulose is mainly the enzymatic conversion of d-fructose by d-allulose 3-epimerase (DAEase). However, the limited specific activity and thermal stability of DAEase restrict its industrial application. Herein, an ultrahigh-throughput screening assay based on the transcription factor PsiR was extensively optimized from the aspects of culture medium components, screening plasmid, and expression host, which enhanced the correction between the fluorescent readout and the enzyme activity. Then, the error-prone PCR (epPCR) library of *Clostridium cellulolyticum* H10 DAEase (CcDAEase) was screened through the above optimized method, and the variant I228V with improved specific activity and thermal stability was obtained. Moreover, after combining two beneficial substitutions, D281G and C289R, which were previously obtained by this optimized assay, the specific activity of the triple-mutation variant I228V/D281G/C289R reached up to 1.42-fold of the wild type (WT), while its half-life (*T*_1/2_) at 60 °C was prolonged by 62.97-fold. The results confirmed the feasibility of the optimized screening assay as a powerful tool for the directed evolution of DAEase.

## 1. Introduction

Rare sugars are considered as potential functional sweeteners due to their low calories and multiple physiological functions. In addition, many rare sugars can be utilized as precursors for medicine production, leading to great market potential in the fields of food and medicine [1]. As a functional low-calorie rare sugar, d-allulose is the C-3 epimer of d-fructose [2], its sweetness is equivalent to 70% of sucrose, while its calorie is only 0.4 kcal g^−1^, which is 90% lower than that of sucrose [3]. The Food and Drug Administration (FDA) has identified d-allulose as generally recognized as safe (GRAS).

d-allulose exists in an extremely small amount in some plants as well as in the heating process of high sugar-containing food [4]. Therefore, environmentally friendly enzymatic preparation is preferred for the industrial production of d-allulose, which is mainly catalyzed by d-allulose 3-epimerase (DAEase) [5]. Since the 1990s, dozens of DAEases from various microbial sources have been characterized [6]. In general, high temperature can accelerate heat and mass transfer, promote the reaction to the direction of d-allulose accumulation, reduce the viscosity of the reaction solution, and inhibit miscellaneous bacterial pollution [7]. Therefore, the industrial production of d-allulose is usually carried out under high temperature condition. Meanwhile, temperature is also a crucial factor affecting the activity of DAEase. In general, the optimum temperatures of different DAEases vary from 40 to 70 °C [6]. However, most DAEases denature rapidly at high temperature due to poor thermal stability, which is difficult to meet the requirements of industrial production. Therefore, gene mining and protein engineering to obtain novel DAEases and their powerful variants with improved performance such as high catalytic activity and robust thermal stability have always been urgent demands for the industrial preparation of d-allulose. 

Protein engineering is an emerging technique to improve the thermal stability of enzymes. Based on the protein structure, rational design is an effective method to modify enzymatic properties. By saturating substrate-recognition sites and introducing disulfide bonds between subunits, Zhu et al. obtained the combined variant Y7H/C66L/I108A/R156C/K260C of *Halanaerobium congolense* DAEase. The half-life (*T*_1/2_) of this variant at 70 °C was 6.3 h and the *T*_m_ value was 79.5 °C, which was 5.2 h and 6.5 °C higher than that of the wild type (WT), respectively [8]. Zhao et al. introduced a disulfide bond into *Clostridium bolteae* DAEase to generate the variant C175/A209C, its *T*_1/2_ at 55 °C was prolonged from 0.37 h of the wild type to 4.5 h [9]. Through sequence alignment analysis and online website-assisted prediction of substrate recognition sites of *C. bolteae* DAEase, Zhang et al. generated the variant Y68I/G109P, which increased *k*_cat_/*K*_m_ by 1.2-fold and *T*_1/2_ from 156 min to 260 min compared to the wild type [10]. B-factor analysis of flexible regions of *Rhodopirellula baltica* DAEase was employed to generate the thermostable variants, and the *T*_m_ of promising variant L144F increased 12.6 °C [11]. Although rational design is universal and efficient, its application is still limited by insufficient understanding of the structure–function relationship, catalytic mechanism, and thermal stability mechanism [12].

Directed evolution is widely utilized in protein engineering to tune enzymatic activity, stability, and stereo-selectivity. Through the construction of the *Agrobacterium tumefaciens* DAEase mutant library by random mutagenesis and high-throughput screening based on detecting the content of substrate d-fructose, Choi et al. successfully obtained variant I33L/S213C with 29.9-fold prolonged *T*_1/2_ at 50 °C [13]. Based on the specific reduction of d-allulose using *Klebsiella pneumoniae* ribitol 2-dehydrogenase (KpRD), Bosshart et al. established a novel 96-well microtiter plate (MTP) screening assay for directed evolution, and successfully increased the *k*_cat_ of engineered thermostable d-tagatose epimerase variant Var8 up to 9-fold [14]. Initial results utilizing directed evolution to enhance the thermal stability of DAEase have been achieved, however, the ultrahigh-throughput assay is the bottleneck that limits the directed evolution of DAEase [15]. The transcription factor PsiR specifically binds to the promoter P*psiA*, inhibiting the transcription of the following reporter gene, while d-allulose can specifically bind with PsiR to terminate its inhibition of transcription [16]. Subsequently, the PsiR-based biosensor was successfully applied for ultrahigh-throughput screening of DAEase variants, eliminating the restriction of screening flux [17].

In this study, further extensive optimization of the above-mentioned PsiR-based screening assay was performed, followed by ultrahigh-throughput screening of the *Clostridium cellulolyticum* DAEase (CcDAEase) error-prone PCR (epPCR) library. A variant with improved specific activity and thermal stability was obtained and extensively characterized. The results demonstrate that the optimized ultrahigh-throughput screening assay is more feasible compared to the original version for the directed evolution of DAEase. 

## 2. Materials and Methods

### 2.1. Materials

The cloning host *Escherichia coli* JM109, expression host *E. coli* BL21 (DE3), recombinant *E. coli* BL21 (DE3)/pET-24a (+)-*kprd* (kpRD represents the gene encoding *Klebsiella pneumonia* ribitol 2-dehydrogenase GenBank accession No. WP_010974118.1) [14], and *E. coli* BL21 (DE3)/pET-20b (+)-*ccdae* (*ccdae* represents the gene encoding CcDAEase, GenBank accession No. ACL75304) [18] were stored in our laboratory. The expression hosts *E. coli* BLR (DE3), *E. coli* HMS174 (DE3), *E. coli* JM109 (DE3), and *E. coli* NovaBlue (DE3) were purchased from Miaolingbio Inc. (Wuhan, China). Plasmid pSB1C3-*psir*-P*psiA*-*mEmerald* [17] was synthesized by Talenbio Co. Ltd. (Shanghai, China).

Antibiotics and lysozyme were bought from Sangon Biotech Co. Ltd. (Shanghai, China). 4-(2-Hydroxyerhyl) piperazine-1-erhanesulfonic acid (HEPES) was purchased from Vita Chemical Reagent Co. Ltd. (Shanghai, China). Chemical reagents including acetonitrile were from Sinopharm Co. (Beijing, China), and d-allulose with a high performance liquid chromatography (HPLC) grade was from Glycarbo Co. Ltd. (Tokyo, Japan). Molecular biology-related reagents and enzymes were purchased from Takara Bio Inc. (Beijin, China), and the Plasmid Extraction Kit and DNA Recovery Kit were from Tiangen Bioch Co. Ltd. (Beijing, China).

### 2.2. Methods

#### 2.2.1. Construction of the Screening Plasmid

The screening plasmid pSB1C3-*psir*-P*psiA*-*mEmerald*-*ccdae* was generated by Megaprimer PCR of whole plasmids (MEGAWHOP) [19]. First, the gene *ccdae* encoding CcDAEase was amplified using pET-20b (+)-*ccdae* as the template and *ccdae*-pSB1C3-F/R as the primer (Table S1). PCR solution (50 μL): 50 ng plasmid template, 25 μL 2× Super Pfx MasterMix, 2 μL forward and reverse primers, and the remaining volume was filled with ddH_2_O. PCR procedure: 94 °C, 4 min; 98 °C 10 s, 56 °C 30 s, 72 °C 40 s, 25 cycles; 72 °C, 10 min. The amplified gene fragment was purified. Second, MEGAWHOP was utilized to construct the recombinant plasmid. The solution: 50 ng pSB1C3-*psir*-P*psiA*-*mEmerald* as the backbone, 25 μL 2× Super Pfx MasterMix, 500 ng *ccdae* as Mega primer, and ddH_2_O up to 50 μL. PCR procedure: 72 °C, 5 min; 98 °C 2 min; 98 °C 30 s, 60 °C 30 s, 72 °C 6 min, 25 cycles; 72 °C, 10 min. The product of MEGAWHOP was incubated with *Dpn* I and directly transformed into the *E. coli* JM109 to obtain the screening plasmid pSB1C3-*psir*-P*psiA*-*mEmerald*-*ccdae*.

#### 2.2.2. Optimization of the Culture Medium

LB medium, TB medium, ZYM-5052 medium [20], and M9 minimal medium were employed as the culture medium for the expression host harboring pSB1C3-*psir*-P*psiA*-*mEmerald*-*ccdae*, respectively. Both the fluorescent values of recombinant *E. coli* BL21 (DE3)/pSB1C3-*psir*-P*psiA*-*mEmerald*-*ccdae* with substrate d-fructose and without d-fructose were measured (TECAN Spark, Tecan Trading AG, Männedorf, Switzerland), respectively. Then, the fluorescent value ratio was calculated, which was the total fluorescent value of cells after fermentation with d-fructose divided by that of cells without d-fructose. When M9 minimal medium was used as the culture medium, an additional 1, 2, 3, and 4 g L^−1^ yeast powder was supplied, respectively, and the corresponding fluorescent values were measured.

#### 2.2.3. Optimization of Osmolyte and Substrate Concentration

Sucrose, glycerol, sorbitol, trehalose, and betaine were utilized during the culture, respectively, to check their effects on the fluorescent value ratio. Different amounts of sorbitol, varying from 10 mmol L^−1^ to 40 mmol L^−1^, were tested for further optimization. Furthermore, during the fermentation, varied concentrations of d-fructose as a substrate were also tested. 

#### 2.2.4. Optimization of Screening Plasmid and Expression Host

The rrnB T1 terminator and T7 Te terminator of pSB1C3-*psir*-P*psiA*-*mEmerald-ccdae* were replaced with the T7 terminator. First, the gene *ccdae* with the T7 terminator was amplified using pET-20b (+)-*ccdae* as the template and *ccdae*-pSB1C3-F and Terminator-R as the primers (Table S1). The amplified fragment was fused to the screening plasmid pSB1C3-*psir*-P*psiA*-*mEmerald-ccdae* through MEGAWHOP to replace the original terminator of *ccdae* gene. The final screening plasmid was denoted as the pSB1C3-*psir*-P*psiA*-*mEmerald-ccdae*-T7 term. Furthermore, different RecA-deficient *E. coli* expression hosts, *E. coli* BLR (DE3), *E. coli* HMS174 (DE3), *E. coli* JM109 (DE3), and *E. coli* NovaBlue (DE3) were tested, respectively. 

#### 2.2.5. Construction of the CcDAEase epPCR Library

The epPCR was performed using the pSB1C3-*psir*-P*psiA*-*mEmerald*-*ccdae*-T7 term as the template and ep-F/R as the primer (Table S1). The solution (50 μL): 10 μL 10× PCR buffer (Mg^2+^ Plus), 50 ng plasmid template, 2 μL ep-F and ep-R, 4 μL dNTP mix, 0.5 μL rTaq, 0.1 mmol L^−1^ Mn^2+^, and the remaining volume was filled with ddH_2_O. PCR procedure: 94 °C, 4 min; 98 °C 10 s, 58 °C 30 s, 72 °C 50 s, 30 cycles; 72 °C, 10 min. The epPCR product was purified and linked with the screening plasmid through MEGAWHOP, then the final product was digested with *Dpn* I and transformed into the expression host to construct the epPCR library.

#### 2.2.6. Ultrahigh-Throughput Screening of the CcDAEase epPCR Library

The ultrahigh-throughput screening assay was modified based on the previously reported method [17]. A total of 1 mL recombinant cells harboring the epPCR library was added to 10 mL LB medium containing 35 µg mL^−1^ chloromycetin (Cm) and cultured at 37 °C and 200 rpm for 8 h. Then, the culture was transferred to the above optimized medium containing 35 µg mL^−1^ Cm with an initial OD_600_ of 0.1, followed by culture at 37 °C and 200 rpm. When OD_600_ reached 0.6, IPTG with a final concentration of 0.2 mmol L^−1^ as well as a final 1000 mmol L^−1^ of d-fructose were added, then the culture was incubated at 37 °C for 1 h. After centrifugation, the cell pellet was collected and washed with PBS buffer (pH 7.4), which was repeated three times. Finally, the cell was diluted with PBS buffer to an OD_600_ value of 0.1. A BD FACSAria Ⅲ (Becton, Dickinson and Company, Franklin Lakes, NJ, USA) with an FITC channel (488 nm excitation, 530/30 BP filter detection) was used to sort the single cell with a fluorescent signal. The top 0.3% cells with the highest fluorescent value of the library were sorted and cultured on agar plates with the respective antibiotic at 37 °C.

#### 2.2.7. Re-Screening of the Sorted epPCR Library

The method constructed by Bosshart et al. [14] was optimized and subsequently utilized for the re-screening of the above-sorted colonies. The colonies were picked for the 96-well MTP containing 100 μL of LB medium with the respective antibiotic in each well. The culture in MTP was cultured at 37 °C and 800 rpm for 8 h and transferred to a new MTP containing 200 μL ZYM-5052 medium with the respective antibiotic for 3 h incubation at 800 rpm. Then, the induction temperature was changed to 25 °C for another 24 h of incubation. The culture solution was centrifuged at 2830× *g* for 15 min, then, the cell pellet was re-suspended with 200 μL HEPES buffer (pH 7.5) containing 1 mmol L^−1^ Co^2+^ as well as 20 μL lysozyme (20 mg mL^−1^). After incubation at 37 °C and 500 rpm for 2 h to release CcDAEase variants, the solution was further incubated at 65 °C for 1 h, and the final crude enzyme solution was obtained by centrifugation at 2830× *g* for 15 min. A total of 20 µL crude CcDAEase variant solution was incubated with 100 µL 200 mmol L^−1^ d-fructose solution at 65 °C and 800 rpm for 30 min, then 120 µL of the KpRD solution containing final concentration of 5.0 mmol L^−1^ NADH was added, and the change in the absorbance value at 340 nm was monitored (TECAN Spark, Tecan Trading AG, Männedorf, Switzerland). 

#### 2.2.8. Expression and Enzymatic Characterization of CcDAEase WT and Variants

The combination of beneficial substitutions was performed by site directed mutation (SDM) [8]. The single colony containing the corresponding plasmid was added to 10 mL of LB medium containing the corresponding antibiotic at 37 °C and 200 rpm for 9 h. A total of 2.5 mL culture was transferred to 50 mL of ZYM-5052 medium with the corresponding antibiotics and cultured at 25 °C and 200 rpm for 24 h. The culture was centrifuged at 11,330× *g* for 15 min, and the cell pellet was re-suspended with HEPES buffer with 0.1 mmol L^−1^ Co^2+^, followed by high-pressure homogenization (800 bar, 2 min, Homogenizer pandaPLUS 2000, GEA Niro Soavi Co., Parma, Italy). Then, the solution was centrifuged at 11,330× *g* for 15 min, and the supernatant containing crude enzyme was passed through a 0.22 μm filter membrane.

A total of 200 μL CcDAEase solution and the pre-heated 800 μL of 100 g L^−1^ d-fructose prepared with HEPES buffer were mixed and reacted accurately at 60 °C for 10 min, then the reaction was immediately terminated by boiling for 10 min. The solution was filtered through 0.22 μm filter membrane filtration and underwent high-performance liquid chromatography (HPLC) for determination. The HPLC condition was: Waters E2695 with refractive index detector (Waters Co., Milford, MA, USA) as well as a ShodexTM AsahipakTM NH2P-50 4E chromatographic column, the column temperature and detection temperature were 35 °C, and the mobile phase was 75% acetonitrile with flow rate of 0.8 mL min^−1^. When the reaction condition was 60 °C and pH 7.5, the amount of enzyme to produce one μmol d-allulose per minute is defined as one enzymatic unit (U).

To determine the optimum pH, the enzyme activities of CcDAEase WT and variants were measured at 60 °C in phosphate buffer (20 mmol L^−1^, pH 5.0–7.0), HEPES buffer (20 mmol L^−1^, pH 7.0–8.0), and Tris-HCl buffer (20 mmol L^−1^, pH 8.0–9.0) with 0.1 mmol L^−1^ Co^2+^, respectively. The relative enzyme activities under corresponding conditions were calculated according to the highest enzyme activity. To measure the pH stability, the crude enzyme solutions of CcDAEase WT and the variants were incubated with corresponding buffer of pH varying from 6.0 to 9.0 at 4 °C for 4 h, respectively. Then, the residual enzyme activities were measured.

To determine the optimum temperature, the enzyme activities of CcDAEase WT and variants at 45, 50, 55, 60, 65, 70, 75, and 80 °C were measured, respectively. The relative enzyme activity under the corresponding condition was presented according to the maximum activity. The crude CcDAEase WT and variants were incubated at 60 °C, and the residual enzyme activities were measured. The relationship between the logarithm of the residual enzyme activity and incubation time was drawn. After linear fitting, the slope of the fitting line was kd (inactivation constant of the enzyme). The *T*_1/2_ of the enzyme at the corresponding temperature was calculated according to the following formula: *T*_1/2_ = ln2/kd [21].

#### 2.2.9. Biosynthesis of d-Allulose Using CcDAEase WT and Variants

For d-allulose biosynthesis, the CcDAEase WT and variants (0.01 mg mL^−1^) were added to 10 mL of 300 g L^−1^ d-fructose solution prepared with 20 mmol L^−1^ HEPES buffer (pH 7.5) containing 0.1 mmol L^−1^ Co^2+^, respectively. Then, the prepared cultures were incubated at 70 °C and 200 rpm. The yield of d-allulose and the residual amount of d-fructose in the reaction solutions were measured by HPLC.

## 3. Results

**Figure 1:**
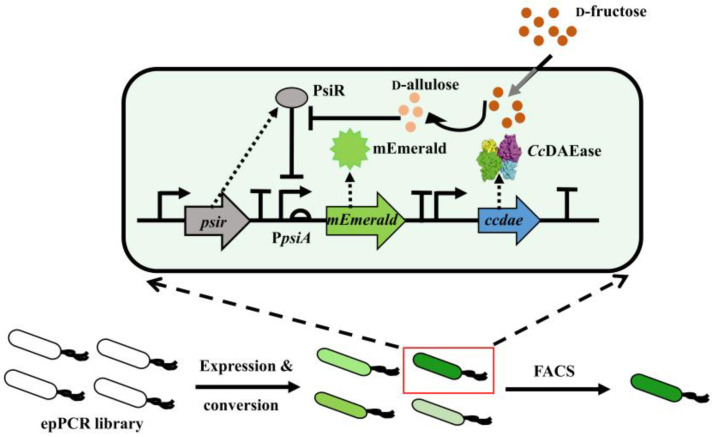
**Scheme of PsiR-based ultrahigh-throughput screening assay.** The *ccdae* epPCR library was transformed into *E. coli* host cells. Then, CcDAEase variants were heterologously expressed in *E. coli*, and the substrate d-fructose was subsequently enzymatically converted to d-allulose, initiating the expression of mEmerald as a reporter. Finally, the recombinant cells exhibiting the highest fluorescent signal were sorted by fluorescence activated cell sorting (FACS).

With the development of biosensors, new light has been shed since the establishment of the ultrahigh-throughput screening assay for the directed evolution of DAEase [17]. The PsiR-based ultrahigh-throughput screening assay is illustrated in Figure 1. The screening plasmid pSB1C3-*psir*-P*psiA*-*mEmerald*-*ccdae* was constructed, and it was confirmed that PsiR and CcDAEase were successfully heterologously expressed in *E. coli* BL21 (DE3) (Figure S1). To further verify whether the fluorescent intensity can be positively coupled with the content of d-allulose, the inducer IPTG as well as 500 mmol L^−1^ of the substrate d-fructose were added during the shake flask fermentation of the recombinant strain *E. coli* BL21 (DE3)/pSB1C3-*psir*-P*psiA*-*mEmerald*-*ccdae*, and the fluorescent value of the strain was measured. The expression conditions were followed exactly as per the previous literature [17], however, the experimental results showed that the fluorescent value with and without adding d-fructose did not change as expected (data not shown). Thus, in order to enhance the coupling of the fluorescent signal and DAEase enzymatic activity, it is quite necessary and urgent to optimize this screening assay to improve the screening efficiency.

### 3.1. Optimization of the Culture Medium

**Figure 2:**
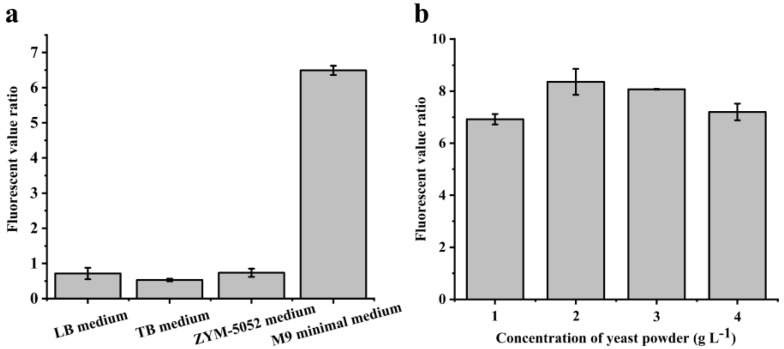
**Optimization of the culture medium for screening.** (**a**) The fluorescent value ratios of *E. coli* BL21 (DE3)/pSB1C3-*psir*-P*psiA*-*mEmerald*-*ccdae* in the LB medium, TB medium, ZYM5052 medium, and M9 minimal medium, respectively. (**b**) The fluorescent value ratios of *E. coli* BL21 (DE3)/pSB1C3-*psir*-P*psiA*-*mEmerald*-*ccdae* in the M9 minimal medium supplemented with different amounts of yeast powder, respectively. All measurements were performed in triplicate, and values are shown as the means ± standard deviation.

First, the influence of the culture medium on the fluorescent signal ratio was explored. The fluorescent values of recombinant cells cultured in different media were detected, and the fluorescent value ratios were calculated, respectively. As shown in Figure 2a, when the LB medium, TB medium, and ZYM-5052 medium were employed as the culture medium, the fluorescent value ratios were 0.7, 0.5, and 0.7, respectively, which might be due to the complex components in the medium negatively affecting the response of the biosensor to d-allulose. Interestingly, the fluorescent value ratio of cells in the M9 minimal medium could reach up to 6.5, which was 9.3-, 13.0-, and 9.3-fold that of the LB medium, TB medium, and ZYM-5052 medium, respectively, indicating the successful coupling of the fluorescent signal with the bioconversion of d-fructose. Therefore, the M9 minimal medium with a relatively simple composition was chosen. 

However, the limited nutrient of the M9 minimal medium slowed down the cell growth. Thus, an appropriate amount of yeast powder was supplied on the basis of the M9 minimal medium to promote the growth of cells. The optimization result is shown in Figure 2b. The fluorescent value ratio first increased and then decreased with the increase in the yeast powder concentration from 1 g L^−1^ to 4 g L^−1^, peaking at 2 g L^−1^. Therefore, the M9 minimal medium with additional 2 g L^−1^ yeast powder was selected as the culture medium.

### 3.2. Optimization of Osmolyte for Promoting the Soluble Expression of CcDAEase

**Figure 3:**
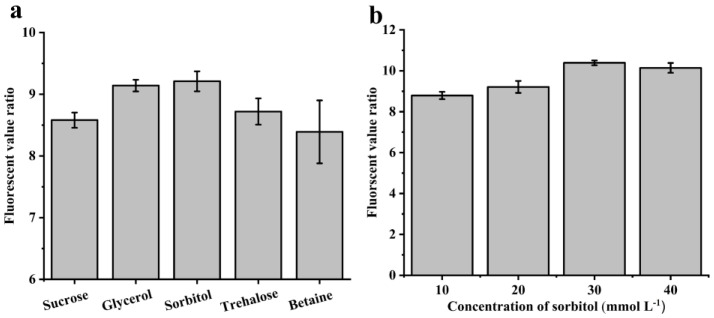
**Optimization of osmolyte in medium.** The fluorescent value ratios of *E. coli* BL21 (DE3)/pSB1C3-*psir*-P*psiA*-*mEmerald*-*ccdae* cultured in medium supplemented with different osmolytes (**a**) and different amounts of sorbitol (**b**). All measurements were performed in triplicate, and values are shown as the means ± standard deviation.

To promote the soluble expression of CcDAEase and further enhance the fluorescent value coupling, different osmolytes were tested. As shown in Figure 3a, the addition of sorbitol exhibited the highest ratio, up to 9.2, among all of the utilized sugars and polyols. Then, the sorbitol concentration was further optimized. When the sorbitol concentration was lower than 30 mmol L^−1^, the fluorescent value ratio gradually increased to 10.4 with the increase in the sorbitol concentration, but decreased with a further increase in the sorbitol concentration (Figure 3b). Therefore, 30 mmol L^−1^ was the optimal concentration of the supplemented sorbitol. Based on the above experimental results, the M9 minimal medium supplemented with 2 g L^−1^ yeast powder and 30 mmol L^−1^ sorbitol was chosen as the final culture medium for further experiments.

### 3.3. Optimization of Substrate d-Fructose Concentration

**Figure 4:**
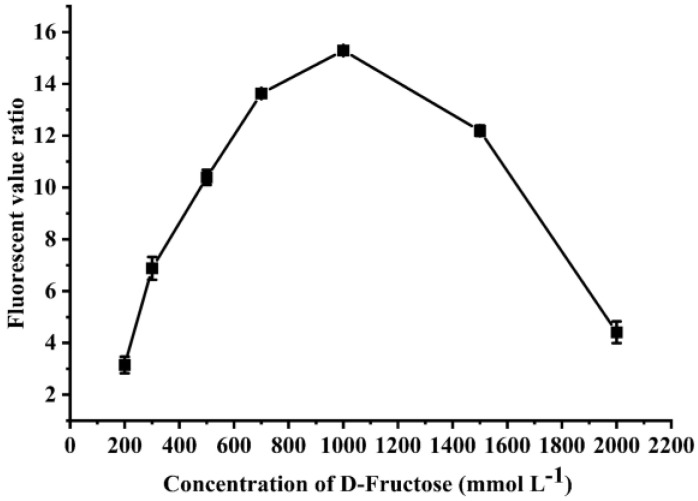
**Optimization of the concentration ofd-fructose as the substrate.**d-fructose with concentration of 200, 300, 500, 700, 1000, 1500, and 2000 mmol L^−1^ were used as the substrate to trigger the fluorescent signal, respectively. All measurements were performed in triplicate, and values are shown as the means ± standard deviation.

To find out the optimal concentration of the substrate, the fluorescent value ratios of cells in the culture medium supplemented with varied concentrations of d-fructose were compared. When the concentration of d-fructose was lower than 1000 mmol L^−1^, the fluorescent value ratio increased with the increase in the d-fructose concentration, and finally peaked at 15.3 (Figure 4). However, when the concentration of d-fructose is higher than 1000 mmol L^−1^, osmotic stress due to a high sugar concentration would negatively affect the growth of bacteria, so the fluorescent value ratio will decrease rapidly [17]. Therefore, 1000 mmol L^−1^ was selected as the substrate concentration in the subsequent experiments.

### 3.4. Optimization of the Screening Plasmid and Expression Host

The optimized culture condition was employed to screen the CcDAEase epPCR library, and FACS was utilized to detect and sort the cells with a high fluorescent signal. However, the results showed that the recombinant cells were mostly concentrated in the non-fluorescent region (data not shown). To uncover the reason for this phenomenon, the recombinant cells without fluorescence were selected and sequenced. The sequencing results demonstrated that the gene fragments encoding mEmerald and CcDAEase were lost. After analyzing the sequence of the screening plasmid, it was found that the terminator sequence of *mEmerald* was completely consistent with that of *ccdae*. Homologous recombination might occur in the process of transformation, resulting in the loss of two gene fragments between the two terminators [22]. 

Therefore, the stability of the screening plasmid was enhanced through the optimization of the screening plasmid and expression host. As shown in Figure S2, the original terminator of the *ccdae* gene in the screening plasmid pSB1C3-*psir*-P*psiA*-*mEmerald-ccdae* was replaced with the T7 terminator through MEGAWHOP, generating the final screening plasmid pSB1C3-*psir*-P*psiA*-*mEmerald-ccdae*-T7 term. In addition, different RecA-deficient *E. coli* expression hosts, *E. coli* BLR (DE3), *E. coli* HMS174 (DE3), *E. coli* JM109 (DE3), and *E. coli* NovaBlue (DE3), were tested to check whether the unexpected recombination could be eliminated. The distribution of fluorescent signals of the recombinant cells was observed by FACS, respectively. Compared to other host cells, the recombinant *E. coli* NovaBlue (DE3) cells showed that the total fluorescent signals were more distributed in the high fluorescent region (Figure S3). Meanwhile, according to the sequencing results, the plasmid was relatively stable in *E. coli* NovaBlue (DE3) (data not shown). Therefore, *E. coli* NovaBlue (DE3) was finally selected as the expression host.

### 3.5. Construction and Ultrahigh-Throughput Screening of the CcDAEase epPCR Library and Enzymatic Characterization of Promising Variant

After completing the above-mentioned optimization, the CcDAEase epPCR library was officially constructed and screened, and the top 0.3% population with the highest fluorescent values was sorted. Furthermore, the potential promising variants were re-screened by the optimized MTP assay [14]. Finally, variant I228V was obtained, and the gene encoding variant I228V was cloned to the expression vector pET-20b (+) and transformed into *E. coli* BL21 (DE3) for heterologous expression and enzymatic characterization. 

The specific activity of the variant I228V was 565.6 U mg^−1^, which was 1.25-fold that of the WT. In addition, the optimal pH and temperature of the variant I228V were determined (Figures S4 and S5). More importantly, after incubation of the crude enzyme at 60 °C for 1 h, the residual activity of I228V was 52.03%, which was higher than that of WT (21.06%) [23], indicating the enhanced thermal stability of variant I228V. To further extensively explore the thermal stability, WT and the variant I228V were incubated at 60 °C, and their residual enzymatic activities were measured at intervals, respectively. The *T*_1/2_ of the variant I228V was 70.51 min at 60 °C, which was 1.85-fold as long as that of the WT (Table 1). The results demonstrated that the specific activity and thermal stability of the variant I228V were successfully improved, which proved the feasibility of the optimized screening assay.

### 3.6. Combination of Beneficial Substitutions to Enhance the Enzymatic Properties of the Variant I228V

To further enhance the properties of the variant I228V, previously identified beneficial substitutions D281G and C289R [23] were combined to generate the triple-mutation variant I228V/D281G/C289R. Then, the enzymatic property of this variant was extensively characterized. The optimal pH of the variant I228V/D281G/C289R was 7.5 (Figure S4), which was a little lower than that of the WT (8.0) [23]. Even the optimal temperatures of the WT and the variant I228V/D281G/C289R were both 65 °C (Figure S5), the *T*_m_ of the variant I228V/D281G/C289R was 83.31 °C, which was 14.4 °C higher than that of WT. In addition, the *T*_1/2_ of this triple-mutation variant at 60, 65, and 70 °C were 2403.60, 672.96, and 226.52 min, respectively (Table 1), which were 62.97-, 73.71-, and 33.71-fold that of the WT, respectively. More importantly, the specific activity of the variant I228V/D281G/C289R was increased to 644.7 U mg^−1^, which was 1.42-fold that of the WT.

Furthermore, to characterize the property of d-allulose preparation, the bioconversion of d-fructose was performed using the WT, the variant I228V, and triple-mutation variant I228V/D281G/C289R, respectively. The final conversion rates of WT, I228V, and I228V/D281G/C289R in 0.01 mg mL^−1^ were 21.64%, 24.67%, and 28.11%, respectively, proving the practical application potential of these promising variants. 

## 4. Discussion 

Independent of the structure–function relationship, directed evolution is an effective and powerful protein engineering strategy to enhance the enzymatic properties [15]. In the procedure of directed evolution, an efficient ultrahigh-throughput screening assay is the crucial factor [24]. To broaden the screening flux of the DAEase library, Armetta et al. successfully developed a biosensor-based assay, which was highly appreciated for the directed evolution of DAEase [17]. However, there is still room for this original system to be further improved. Therefore, in this study, this PsiR-based screening assay was extensively step-by-step optimized to meet the requirements for the protein engineering of DAEase.

Various culture media were tested to check their effects on the fluorescent value ratio of recombinant cells. The M9 minimal medium was determined to be the most suitable among the tested media for fluorescent signal detection. Moreover, yeast powder was added as a supplementary nutrient to support the growth of bacteria due to the poor nutrients in the M9 medium. Meanwhile, the added amount of yeast powder in the M9 minimal medium was optimized to balance the cell growth and signal intensity. 

In general, the screening condition using a biosensor-based assay is under the optimal growth temperature of *E. coli*, 37 °C. However, the enzyme activity of CcDAEase at 37 °C is only 60% of that at its optimal temperature [18]. Therefore, improving the soluble expression of CcDAEase at 37 °C will be one of the challenging and profitable strategies to improve the content of d-allulose, thus increasing the fluorescent value ratio. Meanwhile, previous research has demonstrated that osmolytes can guide the correct folding of protein during translation and improve the soluble expression of protein by maintaining the balance between the osmolyte and protein [25]. Herein, several sugars and polyols with low molecular weight, such as sucrose, glycerol, sorbitol, trehalose, and betaine, were tested. The results confirmed that sorbitol was the most suitable osmolyte to promote fluorescent signal coupling. Moreover, the fluorescent value ratio was essentially affected by the concentration of the enzymatic conversion product d-allulose, and the ratio was also indirectly affected by the concentration of the substrate d-fructose to a certain extent. Therefore, the concentration of substrate in the culture medium was also optimized.

In addition, loss of gene fragment in the screening plasmid was found during the screening procedure. Then, the homologous sequence in the screening plasmid was replaced to reduce the homologous recombination. Meanwhile, RecA is the key protein in the *E. coli* SOS response mechanism to DNA damage, and RecA-deficient *E. coli* strains could reduce the probability of foreign DNA repair and recombination, improving the stability of plasmids [26]. Therefore, different RecA-deficient *E. coli* strains were tested, and the most suitable strain was finally fixed. After the above comprehensive optimization, the fluorescent value ratio of the screening assay was increased from 0.5 to 15.3, which significantly enhanced the correlation between the fluorescent readout and the enzyme activity. 

Furthermore, the feasibility and applicability of the optimized assay were experimentally validated by ultrahigh-throughput screening of the CcDAEase epPCR library. A total of 0.33 million cells were screened by the optimized screening assay, then around 1000 colonies were re-screened by the MTP assay, and the variant I228V with improved specific activity and thermal stability was obtained. The variant I228V from this study showed 1.25-fold that of the specific activity of WT. Meanwhile, I228V also exhibited 1.85-fold of the *T*_1/2_ of WT at 60 °C, demonstrating the enhanced thermal stability. Compared to WT, the weakened steric effect might contribute to the improvement in the thermal stability of variant I228V (Figure S6). Actually, this optimized method was also simultaneously employed in further screening of the CcDAEase epPCR library, and two promising substitutions, D281G and C289R, were successfully obtained [23]. The basic properties of the variant D281G/C289R was also measured, of which the specific activity was 1.90-fold that of the WT, the *T*_m_ was 14.63 °C higher than the WT, and *T*_1/2_ at 60 °C, 65 °C, and 70 °C were 38.24-, 52.00-, and 27.88-fold that of the WT, respectively, indicating the positive contribution of D281G/C289R. Thus, I228V was further combined with D281G and C289R, and the final triple-mutation variant I228V/D281G/C289R exhibited 1.42-fold of specific activity and 62.97-fold of *T*_1/2_ of WT at 60 °C, respectively. In contrast, Armetta et al. obtained the promising variant A142N with 1.15-fold of *k*_cat_/*K*_m_ of CcDAEase WT through the original PsiR-based biosensor [17]. Compared to the original version of this PsiR-based screening assay, the sensitivity of the fluorescent signal was significantly enhanced, and the obtained variant I228V, D281G, and C2289R showed a better performance than the variant A142N generated by the original screening assay, reflecting the improved feasibility and adaptability of the optimized method. Recently, the variant H56R/Q277R/S293R of CcDAEase with enhanced thermal stability was generated through directed evolution utilizing the MTP-based assay, which showed a 9.47-fold improvement in the *T*_1/2_ compared with WT at 60 °C [27]. From the perspective of comparable parameters, the variant I228V/D281G/C289R from this study exhibited better properties than the above-mentioned CcDAEase variants. Therefore, these comprehensive results imply the robust performance and great application potential of the optimized assay in the protein engineering of DAEase. 

In general, a high temperature could result in low viscosity, high reaction speed, and less contamination [7]. Therefore, the biosynthesis of d-allulose was performed at 70 °C, and the final conversion rates of WT, the variant I228V, and I228V/D281G/C289R in 0.01 mg mL^−1^ were 21.64%, 24.67%, and 28.11%, respectively. In addition, the preparation of d-allulose was also performed under the enzyme condition in 0.025 mg mL^−1^, which reached 27.87%, 29.98%, and 31.03%, respectively. Actually, Mu et al. obtained a 29% conversion rate using 750 g L^−1^ d-fructose as the substrate and an excessive 2 g dry cell (wt L^−1^) [18]. Our results demonstrated that sufficient or excessive enzymes can achieve the corresponding conversion rate. Meanwhile, the variant I228V and I228V/D281G/C289R exhibited a higher conversion rate compared to WT with an equal protein concentration, which further proved the industrial application value of the thermal stable variant with high specific activity as well as the feasibility of the above-optimized screening assay. 

In the future, this system can be definitely further optimized to broaden the application range such as the construction of the multi-parameter module to ensure the accuracy of screening [28,29] and the modification of the core promoter, operator, and transcription factor itself to improve the sensitivity [30,31].

## 5. Conclusions

To accelerate the screening efficiency of the directed evolution of DAEase, the PsiR-based ultrahigh-throughput screening assay was successfully optimized, and the fluorescent signal response of the optimized screening assay was significantly enhanced. The applicability of the optimized screening assay was also experimental validated, which broadened the application of biosensors in the further molecular modification of DAEase.

## Figures and Tables

**Table 1:** The *T*_1/2_ of the WT and variants.

Variant	*T*_1/2_ (min)		
	60 °C	65 °C	70 °C
WT *	38.17	9.13	6.72
I228V	70.51	9.34	8.06
I228V/D281G/C289R	2403.60	672.96	226.52

* The data of the WT was from [23].

## Data Availability

All data generated during this study are available in this article and the supplementary materials.

## Supplementary Materials

The following supporting information can be downloaded at: https://www.mdpi.com/article/10.3390/biom12111547/s1. Table S1: Primers used in the study; Figure S1: Construction of pSB1C3-*psir*-P*psiA*-*mEmerald-ccdae* (a) and SDS-PAGE result of PsiR and CcDAEase (b); Figure S2: Replacement of the terminator for the screening plasmid; Figure S3: Distribution of the fluorescent signals of different expression hosts containing the screening plasmid; Figure S4: Optimal pH of CcDAEase wild-type (WT), I228V, and I228V/D281G/C289R; Figure S5: Optimal temperature of WT, I228V, and I228V/D281G/C289R; Figure S6: The structure-function analysis of CcDAEase WT (a) and variant I228V (b). ref [32].
