# Supplementary material for: Optimization of Ultrahigh-Throughput Screening Assay for Protein Engineering of d-Allulose 3-Epimerase
Source: Biomolecules. 2022 Oct 24;12(11):1547. doi: 10.3390/biom12111547 (PMC9687117; doi:10.3390/biom12111547)
Supplement: Supplementary file 1 [file biomolecules-12-01547-s001.zip › biomolecules-1934750-supplementary.pdf]

Optimization of ultrahigh-throughput screening assay for protein engineering of D-allulose 3-epimerase

Supplementary materials

Zhanzhi Liu[1,2,3], Shuhan Liu[1,2,3], Jingyi Jia[1,2,3], Liuxin Wang[2], Feng Wang[2], Xiaoyue Pan[2], Jing Wu[1,2,3], Sheng Chen[1,2,3,*]

1 State Key Laboratory of Food Science and Technology, Jiangnan University, 1800 Lihu Avenue, Wuxi, 214122, Jiangsu Province, China

2 Key Laboratory of Industrial Biotechnology Ministry of Education, School of Biotechnology, Jiangnan University, 1800 Lihu Avenue, Wuxi, 214122, Jiangsu Province, China

3 International Joint Laboratory on Food Safety, Jiangnan University, 1800 Lihu Avenue, Wuxi, 214122, Jiangsu Province, China

* Corresponding author:

chensheng@jiangnan.edu.cn

Table S1 Primers used in the study

Primer	Sequence (5'-3')
ep-F	ATGAAACATGGCATCTATTA
ep-R	TGGTGGTGGTGGTGCTCGAG
ccdae-pSB1C3-F	AGAAACTCGAGGATGAAACATGGCATCTAT
ccdae-pSB1C3-R	CCTGGGCATGCCGCTTCAGTGGTGGTGGTG
Terminator-R	TAGTTCTGCTATAAACAAAAAACCCCTCAA
D281G-SDM-F	GCACAGGCAGCCTTAGGTTTTTCACGCTATGTG
D281G-SDM-R	CACATAGCGTGAAAAACCTAAGGCTGCCTGTGC
C289R-SDM-F	CGCTATGTGTTAGAACGTCATAAACATAGCCTC
C289R-SDM-R	GAGGCTATGTTTATGACGTTCTAACACATAGCG

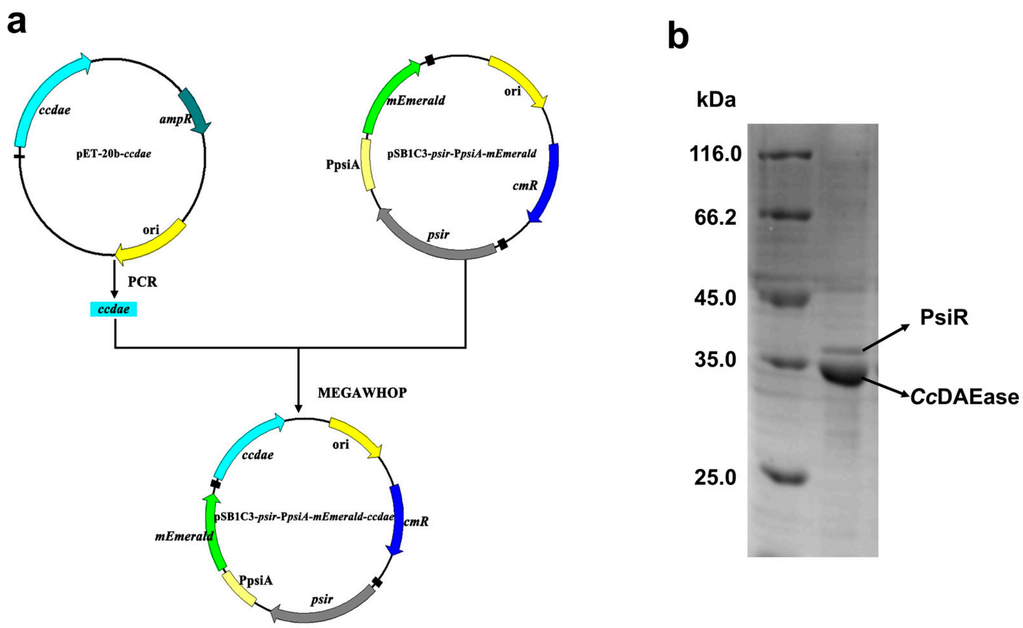

Figure S1 Construction of pSB1C3-*psir*-P*psiA*-*mEmerald*-*ccdae* (a) and SDS-PAGE result of PsiR and CcDAEase (b). The screening plasmid pSB1C3-*psir*-P*psiA*-*mEmerald*-*ccdae* was transformed into the expression host *E. coli* BL21 (DE3), and the recombinant strain was cultured for 24 h, then the heterologous expression result was checked by SDS-PAGE. As shown in Fig. S1b, there were two obvious bands at 39.0 kDa and 33.0 kDa, which were consistent with the theoretical protein size of PsiR and CcDAEase, respectively, confirming the successful expression of PsiR and CcDAEase.

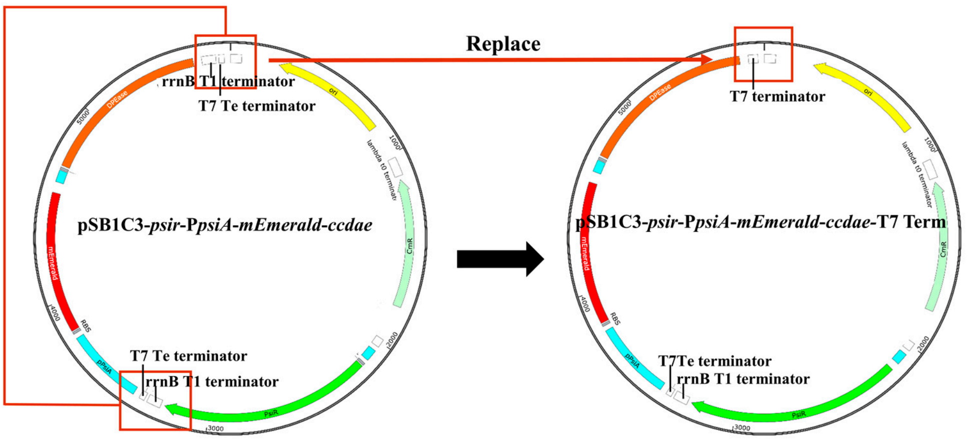

Figure S2 Replacement of the terminator for the screening plasmid. The rrnB T1 terminator and T7 Te terminator of pSB1C3-*psir*-P*psiA*-*mEmerald*-*ccdae* were replaced with T7 terminator, and the final screening plasmid was denoted as pSB1C3-*psir*-P*psiA*-*mEmerald*-*ccdae*-T7 Term. The utilized primers were *ccdae*-pSB1C3-F and Terminator-R listed in Table S1.

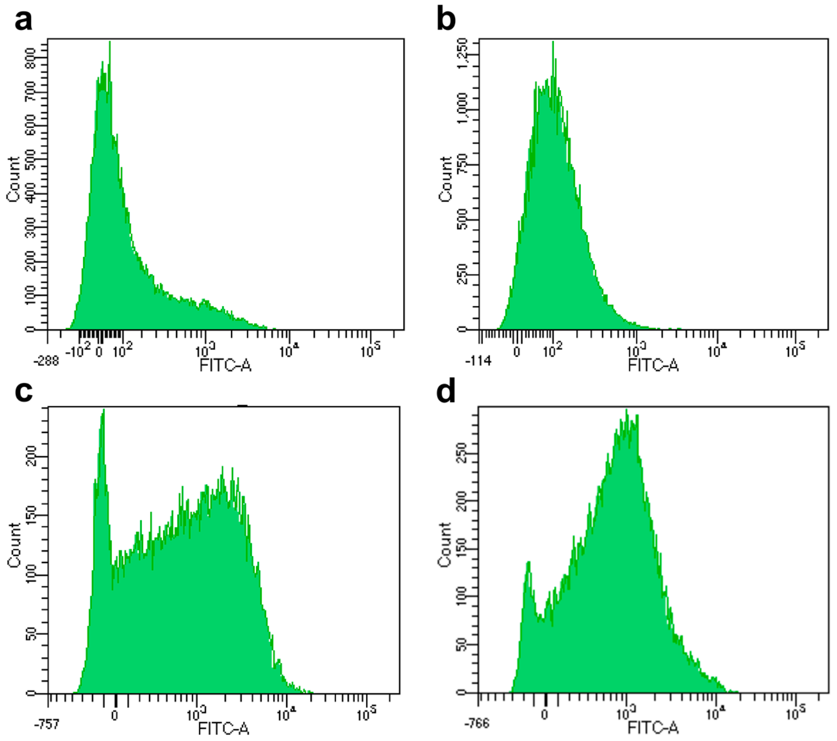

Figure S3 Distribution of the fluorescent signals of different expression hosts containing the screening plasmid. Using *E. coli* BLR (DE3) (a), *E. coli* HMS174 (DE3) (b), *E. coli* JM109 (DE3) (c), and *E. coli* NovaBlue (DE3) (d) as expression host, respectively.

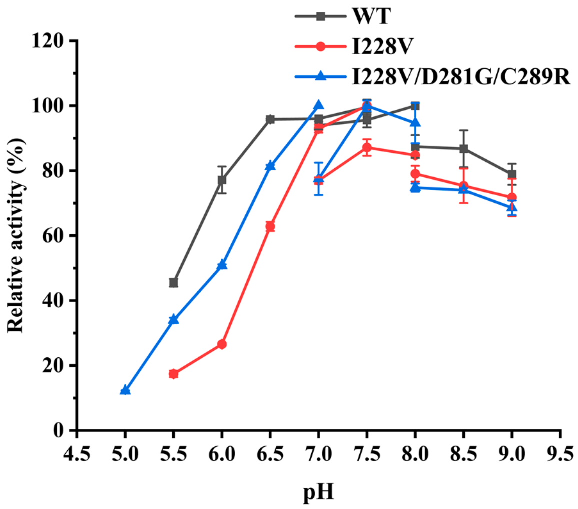

Figure S4 Optimal pH of CcDAEase wild-type (WT), I228V, and I228V/D281G/C289R. The optimal pH of WT, I228V, and I228V/D281G/C289R were 8.0, 7.5, and 7.5 respectively. All measurements were performed in triplicate, and values were shown as means ± standard deviation. The data of CcDAEase WT was from Liu et al. [1].

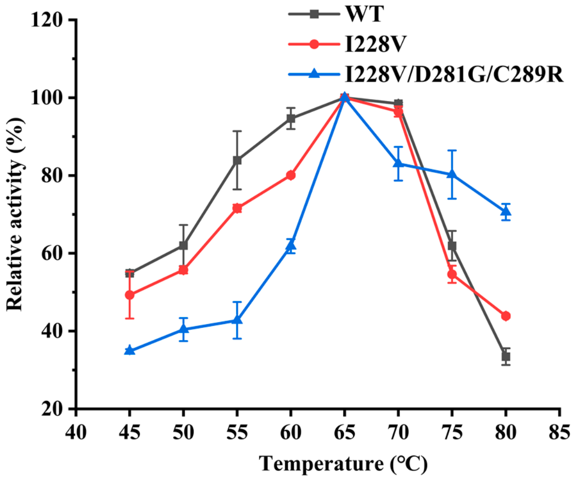

Figure S5 Optimal temperature of WT, I228V, and I228V/D281G/C289R. The optimal temperature of WT, I228V, and I228V/D281G/C289R were 65 ℃. All measurements were performed in triplicate, and values were shown as means ± standard deviation. The data of CcDAEase WT was from Liu et al. [1].

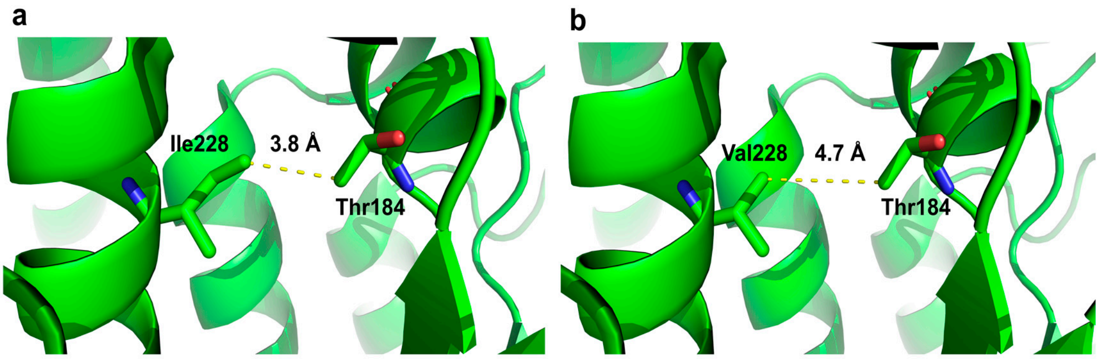

Figure S6 The structure-function analysis of CcDAEase WT (a) and variant I228V (b). The distance between Ile228 and Thr194 of WT was 3.8 Å, and the distance between Val228 and Thr194 of variant I228V was 4.7 Å. Based on the structure of WT (PDB: 3VNK) [2], the variant I228V structure was simulated by Pymol software.

Reference

1. Liu, S.; Wang, Y.; Kong, D.; Wu, J.; Liu, Z. Enhancing the thermostability of D-allulose 3-epimerase from *Clostridium cellulolyticum* H10 via directed evolution. *Syst. Microbiol. and Biomanuf.* **2022,** *2*, 685-694.
2. Chan, H.; Zhu, Y.; Hu, Y.; Ko, T.; Huang, C.; Ren, H.; Chen, C.; Ma, Y.; Guo, R.; Sun, Y. Crystal structures of D-psicose 3-epimerase from *Clostridium cellulolyticum* H10 and its complex with ketohexose sugars. *Protein Cell* **2012,** *3*, 123-131.
